# Nodule Regression in Adults With Nodular Gastritis

**DOI:** 10.14740/gr692w

**Published:** 2015-12-31

**Authors:** Ji Wan Kim, Sun-Young Lee, Jeong Hwan Kim, In-Kyung Sung, Hyung Seok Park, Chan-Sup Shim, Hye Seung Han

**Affiliations:** a Department of Internal Medicine, Konkuk University School of Medicine, Seoul, Korea; b Department of Pathology, Konkuk University School of Medicine, Seoul, Korea

**Keywords:** *Helicobacter pylori*, Eradication, Gastritis, Nodule, Regression

## Abstract

**Background:**

Nodular gastritis (NG) is associated with the presence of *Helicobacter pylori* infection, but there are controversies on nodule regression in adults. The aim of this study was to analyze the factors that are related to the nodule regression in adults diagnosed as NG.

**Methods:**

Adult population who were diagnosed as NG with *H. pylori* infection during esophagogastroduodenoscopy (EGD) at our center were included. Changes in the size and location of the nodules, status of *H. pylori* infection, upper gastrointestinal (UGI) symptom, EGD and pathology findings were analyzed between the initial and follow-up tests.

**Results:**

Of the 117 NG patients, 66.7% (12/18) of the eradicated NG patients showed nodule regression after *H. pylori* eradication, whereas 9.9% (9/99) of the non-eradicated NG patients showed spontaneous nodule regression without *H. pylori* eradication (P < 0.001). Nodule regression was more frequent in NG patients with antral nodule location (P = 0.010), small-sized nodules (P = 0.029), *H. pylori* eradication (P < 0.001), UGI symptom (P = 0.007), and a long-term follow-up period (P = 0.030). On the logistic regression analysis, nodule regression was inversely correlated with the persistent *H. pylori* infection on the follow-up test (odds ratio (OR): 0.020, 95% confidence interval (CI): 0.003 - 0.137, P < 0.001) and short-term follow-up period < 30.5 months (OR: 0.140, 95% CI: 0.028 - 0.700, P = 0.017).

**Conclusions:**

In adults with NG, *H. pylori* eradication is the most significant factor associated with nodule regression. Long-term follow-up period is also correlated with nodule regression, but is less significant than *H. pylori* eradication. Our findings suggest that *H. pylori* eradication should be considered to promote nodule regression in NG patients with *H. pylori* infection.

## Introduction

Nodular gastritis (NG) is characterized by an unusual goose-flesh appearance on esophagogastroduodenoscopy (EGD). There is accumulating evidence that *Helicobacter pylori* infection plays a role in the pathogenesis of NG [1-3]. Most cases are diagnosed incidentally because they are asymptomatic [4], but some patients manifest atypical upper gastrointestinal (UGI) symptoms such as indigestion, nausea, abdominal bloating, epigastric pain or abdominal discomfort [5, 6]. However, there is insufficient evidence to recommend a *H. pylori* test-and-treat strategy as an initial management approach to Korean adults presenting as functional dyspepsia [6]. The prevalence of NG decreases with aging, and is higher in women than in men [2, 4, 7]. We found that the prevalence of NG in Korean adults was 52 (2.0%) of 2,059 asymptomatic Korean adults [4].

There are two endoscopic patterns of nodules in NG, and the clinicopathological findings do not appear to differ significantly between the two patterns [7]. A granular-type consists of small nodules with a size < 5 mm exhibiting regular protrusion, while a nodular-type consists of larger nodules (≥ 5 mm) exhibiting large uniform protrusions. With regard to the location of the nodules, NG can involve both the antrum and body, with a predominantly nodular-type pattern on the antrum [1, 3]. In addition, the nodules on the antrum change from the diffuse-type NG to the nondiffuse-type NG with the progress of gastric atrophy [8].

The prognosis differs in adults with NG; some might progress to a diffuse-type gastric cancer and a few may progress to a lymphofollicular malignancy [9, 10]. Several studies have found reductions in symptoms and in the risk of peptic ulcers and possibly gastric cancer following *H. pylori* eradication [5, 11, 12]. Nevertheless, the characteristics of NG patients who show nodule regression are still uncertain. The aim of the present study was to identify the factors related to the improvement of NG in adults with *H. pylori* infection.

## Patients and Methods

### Study population

This cross-sectional study included Korean adults who were diagnosed as NG with *H. pylori* infection at our center between August 2005 and April 2013. NG patients were included when EGD was followed up at our center after the initial diagnosis. NG patients were excluded when the age was below 20 years old, had a history gastric surgery, or had other significant disease at the time of diagnosis. Furthermore, those without preserved gastric biopsied specimens were also excluded from the study.

All of the included NG patients gave their written informed consent before EGD and answered to the questionnaires on their recent symptoms and medical history. This study was approved by the Institutional Review Board (IRB) of Konkuk University School of Medicine which confirmed that the study was in accordance with the ethical guidelines of the Helsinki Declaration. After the IRB approval at our center (KUH 1010574), it was registered as ClinicalTrials.gov ID: KCT0001148 (https://cris.nih.go.kr).

### Endoscopic examination

EGD was performed with either EG-2990i endoscope (Pentax, Tokyo, Japan) or GIF-H260 endoscope (Olympus, Tokyo, Japan) at our center. Endoscopic findings of the background mucosa was classified as normal finding, chronic superficial gastritis, hemorrhagic gastritis, erosive gastritis, verrucous gastritis, chronic atrophic gastritis, metaplastic gastrtiis, and hypertrophic gastririts. Other notable findings such as nodular changes or hemorrhagic spots were recorded additionally.

### Diagnosis for NG

NG was defined as diffuse chicken-skin-like mucosal changes or unusual goose-flesh apprearance on the endoscopic finding. When the nodules exhibit regular protrusion with a size < 5 mm, NG was subclassified as a small granular-type. When the nodules exhibit larger size with uniform protrusion, it was subclassified as a large nodular-type.

### Histopathology

Gastric biopsied specimens were fixed in formalin. They were stained with hematoxylin and eosin (H&E) and with modified Giemsa staining. Positive Giemsa staining was regarded as *H. pylori* infection. Based on the updated Sydney system, the grades were scored as either none, mild, moderate, or marked for chronic changes including atrophy and intestinal metaplasia (0 = none, 1 = mild, 2 = moderate, and 3 = marked infiltration). In addition, the grades were scored for acute changes including activity (the intensity of acute polymorphonuclear cell infiltrates) and inflammation (the intensity of chronic mononuclear cell infiltrates).

### Diganosis and treatment for *H. pylori* infection

The presence of *H. pylori* infection was determined when more than one of the invasive tests showed positive finding. For the invasive tests, histology and Giemsa stain were performed during EGD with or without the rapid urease test (CLO test; Roswell, USA manufactured by Korea Green Cross Medical Science, Eumseong, Korea).

NG patients were sent to the Digestive Disease Center if the patient agreed on further management. For the treatment, *H. pylori* eradication was performed using a triple therapy with a proton pump inhibitor (PPI, standard dose twice), amoxicillin (1 g twice) and clarithromycin (500 mg twice) daily for 1 week according to the guideline [13]. If the initial eradication has failed, a quadrauple therapy was performed with a PPI (standard dose twice), bismuth (120 mg four times), tetracycline (500 mg four times), and metronidazole (500 mg thrice) daily for 1 week.

### Follow-up tests

After the initial diagnosis, NG patients underwent EGD and *H. pylori* test at our center as the initial tests. Changes in the size and location of the nodules, status of *H. pylori* infection, UGI symptom, EGD and pathology findings were compared with the previous findings by one gastroenterologist (Dr. J. W. Kim) to exclude any interobserver variability. Improvement of NG was defined as nodule regression on EGD findings.

### Statistical analysis

Continuous variables were analyzed using the Student’s *t*-test and were expressed as the mean ± standard deviation (SD) values. Variables exhibiting skewed distributions were expressed as median values with ranges (minimum and maximum values), and differences were tested using the Kruskal-Wallis test. Categorical variables were analyzed using the Chi-square test and were presented as percentage. For the cut-off value of follow-up periods, a receiver operating characteristic (ROC) curve was constructed by plotting sensitivity (true-positive rate) against 1-specificity (false-positive rate) over all possible threshold levels of follow-up periods that were found to be related to the nodule regression. Based on the area under the ROC curve (AUC) analysis with 95% confidence interval (CI) and standard error (SE) values, the accuracy of the follow-up period for detecting nodule regression was caluculated. With the cut-off value provided by the ROC curve analysis, logistic regression analysis was performed to identify variables that were associated with nodule regression. Odds ratio (OR) and 95% CI were analyzed for each significant variable. All analyses were performed using SPSS software, Windows version 19.0. A P-value of less than 0.05 was considered to be statistically significant.

## Results

### Baseline characteristics of the NG patients

A total of 117 NG patients fulfilled the study criteria and underwent follow-up EGD at our center after the initial diagnosis of NG. Most of the NG patients (85.5%) did not have UGI symptom at the time of endoscopic examination (Table 1).

**Table 1:** Baseline Characteristics of the Included Subjects

Characteristics	NG patients (n = 117)
Age (years, mean ± SD)	38.3 ± 8.0
Gender (female, %)	86 (73.5%)
Follow-up period (months, mean ± SD)	32.0 ± 14.7
Presence of UGI symptom (%)	
Epigastric pain	8 (6.8%)
Indigestion	5 (4.3%)
Abdominal distention	2 (1.7%)
Nausea	2 (1.7%)
Size of the nodules (%)	
Small granular-type	64 (54.7%)
Large nodular-type	53 (45.3%)
Location of the nodules (%)	
Antrum only	73 (62.4%)
Body involved	44 (37.6%)
Presence of hemorrhagic spots (%)	21 (17.9%)
*H. pylori* eradication (%)	18 (15.4%)
Pathology based on the updated Sydney system	
Inflammation (none/mild/moderate/marked)	0:5:91:21
Activity (none/mild/moderate/marked)	5:42:61:9
Atrophy (none/mild/moderate/marked)	91:22:4:0
Intestinal metaplasia (none/mild/moderate/marked)	106:10:0:1
*H. pylori* (none/mild/moderate/marked)	6:10:48:53

SD: standard deviation; UGI: upper gastrointestinal.

### Follow-up tests with or without *H. pylori* eradication

Of the included NG patients, 18 (15.4%) underwent *H. pylori* eradication after the diagnosis. At the follow-up tests, none of the eradicated patients showed *H. pylori* infection, whereas all of the non-eradicated patients showed persistent *H. pylori* infection. Nodule regression was noticed at the follow-up tests in 66.7% of the 18 eradicated NG patients after *H. pylori* eradication, whereas it was noticed in 9.9% of the 99 non-eradicated NG patients (P < 0.001). None of these patients progressed to gastric cancer during the follow-up period.

### Variables correlated with nodule regression

Using the correlation analysis, follow-up period (r = -0.200, P = 0.030), UGI symptom (r = -0.250, P = 0.007), nodule size (r = 0.202, P = 0.029) and *H. pylori* eradication (r = -0.541, P < 0.001) were related to nodule regression (Table 2). When the patients were subclassified into two groups according to the presence of nodules at the follow-up tests, nodule regression was related to the presence of UGI symptom (P = 0.007), *H. pylori* eradication (P < 0.001), and small size (P = 0.029) of the nodules (Table 3).

**Table 2:** Correlation Analysis of the Variables on Nodule Regression

Variables	Correlation coefficient	P-value
Age	0.044	0.637
Gender	0.072	0.437
Follow-up period	-0.200	0.030
Presence of upper gastrointestinal symptom	-0.250	0.007
Size of the nodules	0.202	0.029
Location of the nodules	0.133	0.152
Presence of hemorrhagic spots	0.103	0.271
*H. pylori* eradication	-0.541	< 0.001
Pathology findings based on the updated Sydney system		
Inflammation	0.142	0.127
Activity	-0.088	0.345
Atrophy	-0.027	0.773
Intestinal metaplasia	-0.153	0.099
*H. pylori* infiltration	-0.012	0.899

**Table 3:** Comparison Between the Patients With Nodule Regression and Those With Persistent Nodules at the Follow-Up Tests

Variables	Patients with nodule regression (n = 21)	Patients with persistent nodules (n = 96)	P-value
Age (years, mean ± SD)	37.6 ± 8.5	38.5 ± 7.9	0.637
Gender (female, %)	14 (66.7%)	72 (75.0%)	0.433
Presence of UGI symptom (%)	7 (33.3%)	10 (10.4%)	0.007
Size of the nodules (%)			0.029
Small granular-type	16 (76.2%)	48 (50.0%)	
Large nodular-type	5 (23.8%)	48 (50.0%)	
Location of the nodules (%)			0.214
Antrum only	16 (76.2%)	57 (59.4%)	
Body involved	5 (23.8%)	39 (40.6%)	
Presence of hemorrhagic spots (%)	2 (9.5%)	19 (19.8%)	0.267
Pathology scores (0:1:2:3)			
Inflammation	0:2:17:2	0:3:74:19	0.260
Activity	0:7:12:2	5:35:49:7	0.711
Atrophy	17:2:2:0	74:20:2:0	0.136
Intestinal metaplasia	18: 2:0:1	88:8:0:0	0.097
*H. pylori*	1:1:10:9	5:9:38:44	0.864
*H. pylori* eradication (%)	12 (57.1%)	6 (6.2%)	< 0.001
Follow-up period (months, mean ± SD)	38.2 ± 12.1	30.6 ± 14.9	0.030

SD: standard deviation; UGI: upper gastrointestinal.

Furthermore, NG patients with nodule regression showed a longer follow-up period (38.2 ± 12.1 months) than those without nodule regression (30.6 ± 14.9 months, P = 0.030). Using the ROC analysis, the cut-off value of follow-up period on nodule regression was 30.5 months (AUC: 0.651, 95% CI: 0.540 - 0.763, SE: 0.057, P = 0.030) with a sensitivity of 71.4% and specificity of 53.1%.

### Importance of *H. pylori* eradication and follow-up period on nodule regression

Of all significant variables, only *H. pylori* eradication and follow-up period were significant on the logistic regression analysis (Table 4). Nodule regression was inversely correlated with the persistent *H. pylori* infection on the follow-up test (OR: 0.020, 95% CI: 0.003 - 0.137, P < 0.001) and short-term follow-up period < 30.5 months (OR: 0.140, 95% CI: 0.028 - 0.700, P = 0.017). Conversely, persistent nodules in NG patients were significantly higher in the non-eradicated group (OR: 49.948, 95% CI: 7.274 - 342.957, P < 0.001) and short-term follow-up period < 30.5 months (OR: 7.132, 95% CI: 1.430 - 35.581, P = 0.017).

**Table 4:** Significant Variables Related to Nodule Regression

Variables	Odds ratio	95% confidence interval	P-value
*H. pylori* eradication			
No (persistent *H. pylori* infection)	0.020	0.003 - 0.137	< 0.001
Yes	1 (reference)		
Follow-up period*			
< 30.5 months	0.140	0.028 - 0.700	0.017
≥ 30.5 months	1 (reference)		
Upper gastrointestinal symptom			
Present	1.483	0.246 - 8.936	0.667
Absent	1 (reference)		
Size of the nodules			
Small	2.670	0.738 - 9.658	0.134
Large	1 (reference)		

*Cut-off value was determined by the ROC curve analysis.

### Subgroup analysis according to *H. pylori* eradication

Of 99 NG patients without eradication, nine (9.1%) showed spontaneous nodule regression. Between these nine NG patients with nodule regression and other 90 NG patients with persistent nodules, there were no significant differences on age (P = 0.605), gender (P = 0.224), nodule size (P = 0.165), location (P = 0.279), UGI symptom (P = 0.502), hemorrhagic spots (P = 0.351), and follow-up period (P = 0.131). With regard to the scores based on the updated Sydney system, there were no significant differences on inflammation (P = 0.351), activity (P = 0.824), atrophy (P = 0.094), and *H. pylori* infiltration (P = 0.880) scores, but median intestinal metaplasia score was significantly higher in those with spontaneous nodule regression (0.25 ranging from 0 to 3) than those without regression (0.08 ranging from 0 to 1, P = 0.008).

All the eradicated patients exhibited no *H. pylori* at the follow-up tests, and 12 (66.7%) showed nodule regression. Between these 12 NG patients with nodule regression and other six NG patients with persistent nodules, there were no significant differences on age (P = 0.944), gender (P = 0.344), nodule size (P = 0.294), location (P = 0.073), UGI symptom (P = 0.563) and hemorrhagic spots (P = 0.569). Mean follow-up period of the 12 improved NG patients (37.6 ± 12.6 months) was significantly longer than that of the six persistent NG patients (21.8 ± 6.5 months, P = 0.012). On the pathology findings, there were no significant differences on activity (P = 0.258), atrophy (P = 0.201), intestinal metaplasia (P = 0.621), and *H. pylori* infiltration (P = 0.285), but mean inflammation score was significantly higher in NG patients with persistent nodules (2.3 ± 0.5) than in those with nodule regression (2.0 ± 0.0, P = 0.035).

## Discussion

This study found that *H. pylori* eradication is the most important factor related to nodule regression in NG. In addition, longer follow-up period was a significant independent factor for nodule regression in NG patients with *H. pylori* eradication. Neither the gender nor the age of the patient was related to the prognosis of NG, despite NG being more common in young women.

In this study, two-thirds of the eradicated NG patients exhibited complete improvement of NG at the follow-up tests. Similarly, endoscopic evidence of NG disappeared in 63.2% of the NG patients at 2 months after *H. pylori* eradication in a Chinese study [11]. The observation of nodule regression after eradication in the present study supports the recommendation that *H. pylori* eradication should be performed to the adults with NG for nodule regression [5, 11, 12]. Notably, such improvements do not occur immediately, and regression requires a substantial amount of time [8]. This study exhibited that longer follow-up period is also correlated with the improvement of NG, but follow-up period was less significant than eradication. Our findings suggest that longer follow-up period is a significant independent factor for nodule regression in NG patients, but it is less accurate when used alone for predicting nodule regression without eradication.

Interestingly, 9.1% of the NG patients without *H. pylori* eradication in the present study exhibited spontaneous regression of the nodules, even with persistent *H. pylori* infection. Few studies have found a tendency for antral nodules to regress more frequently than those on the body [1, 3]. This finding may be attributable to differences in the severity and depth of *H. pylori*-induced inflammation on the antrum and body, and because antral nodules tend to regress in association with atrophic changes [8, 14]. Although the correlation between NG and UGI symptom is still uncertain [6], nodule regression was more frequent in NG patients with UGI syptoms in our study, because those with symptoms tend to undergo *H. pylori* eradication. The presence of UGI symptom was not an idependent signficant factor for nodule regression in this study.

With regard to nodule size, small ones (granular-type NG) tended to regress more often than the large ones (nodular-type NG) in our NG patients. This can be explained by the histological findings, whereby smaller lymphoid follicles resolve more easily than large lymphoid follicles with other inflammatory cells. Although we were unable to determine why the smaller antral nodules regressed more readily than the large body nodules, we suggest that this is due to the different proportions of histopathological features, including the presence of gastric atrophy, intestinal metaplasia, lymphoid aggregates, or eosinophil infiltrates in the mucosal layer, as shown previously [15]. The histopathological features of NG differ according to the size and location of the nodules, and thus improvement after eradication will differ based on these factors.

This study found that pathology findings could be related to nodule regression. In case of eradicated NG patients, those with persistent nodules even after the eradication exhibited higher inflammation score. Our findings suggest that higher intensity of chronic mononuclear cell infiltrates in these NG patients might interfere nodule regression by continous aggregation of the lymphoid follicles. On the other hand, in case of non-eradicated NG patients, those with spontaneous nodule regression exhibited higher intestinal metaplasia score. This is consistent with previous studies that NG improves upon senile changes of the gastric mucosa such as atrophic gastriits and intestinal metaplasia [7-9, 14].

There are several limitations in this study. Firstly, *H. pylori* eradication was not performed in all of the NG patients, because it is not covered by insurance in Korea. It was performed only in NG patients who agreed on additional payment for eradication. Secondly, there was no malignant change in all NG patients at the follow-up tests. Therefore, we could not find a correlation between NG and gastric malignancy in this study. Despite all these limitations, there was a significant difference on nodule regression between the NG patients with *H. pylori* eradication and those without eradication.

### Conclusions

*H. pylori* eradication is the most significant factor associated with the prognosis of NG in adults. Although there is a tendency for NG patients with longer follow-up to regress nodules, the significance as a prognostic factor for nodule regression is low when compared to that of *H. pylori* eradication. Together, the findings of this study suggest that adults with NG should undergo *H. pylori* eradication to promote nodule regression regardless of the size and location of the nodules.
